# Association of Ultrafiltration Rate with Structural Remodeling of the Left Heart and Arrhythmias in Hemodialysis Patients: A Cross-Sectional Observational Study

**DOI:** 10.3390/clinpract16100182

**Published:** 2026-09-30

**Authors:** Ammar Brkić, Kenana Ljuca, Emir Tulumović, Nadina Ljuca, Nermin Salkić, Emir Bećirović, Amir Tursunović, Minela Bećirović, Bahrudin Hadžiefendić

**Affiliations:** 1 Department of Intensive Care, Clinic for Internal Medicine, University Clinical Center Tuzla, 75000 Tuzla, Bosnia and Herzegovina; ammar.brkic@ukctuzla.ba (A.B.); becirovic.emir@live.com (E.B.); 2 Department of Gynecology and Obstetrics, University Clinical Center Ljubljana, 1000 Ljubljana, Slovenia; 3 Department of Pharmacology, Faculty of Medicine, University of Tuzla, 75000 Tuzla, Bosnia and Herzegovina; ljucanadina@gmail.com; 4 Department of Gastroenterology, Clinic for Internal Medicine, University Clinical Center Tuzla, 75000 Tuzla, Bosnia and Herzegovina; emir.tulumovic@ukctuzla.ba; 5 Erbe Elektromedizin GmbH, 72072 Tuebingen, Germany; 6 Department of Surgery, University Clinical Center Tuzla, 75000 Tuzla, Bosnia and Herzegovina; tursunovicamirmd@gmail.com; 7 Department of Nephrology and Hemodialysis, Clinic for Internal Medicine, University Clinical Center Tuzla, 75000 Tuzla, Bosnia and Herzegovina; minela03@live.com; 8 Department of Surgery, Eurofarm Center Policlinic Tuzla, 75000 Tuzla, Bosnia and Herzegovina; batohadziefendic@gmail.com

**Keywords:** hemodialysis, ultrafiltration rate, cardiac morphological changes, arrhythmias

## Abstract

**Background/aim:** Hemodialysis patients face a high burden of cardiovascular diseases, which account for over 40% of mortality in this population. In these patients, approximately two-thirds of cardiovascular deaths are due to arrhythmia. The aim of this study was to determine whether the ultrafiltration rate is associated with morphological and functional parameters of the left heart and arrhythmias. **Methods:** This work was a cross-sectional observational clinical study, with 75 hemodialysis patients. Patients were divided into two groups based on ultrafiltration rate (UFR): higher-UFR group, ultrafiltration rate ≥ 10 mL/kg/h (*n* = 37); lower-UFR group, ultrafiltration rate < 10 mL/kg/h (*n* = 38). All patients were analyzed for myocardial morphological and functional parameters and arrhythmias. **Results:** Statistically significant higher values were observed for the left atrium diameter (LAD) (47.56 ± 4.82 vs. 44.75 ± 2.33 mm, *p* = 0.0019), the left atrial volume index (LAVI) (43.28 ± 7.13 vs. 36.43 ± 8.52 mL/m^2^, *p* = 0.0003), and the left ventricular mass index (LVMI) (138.47 ± 29.32 vs. 116.04 ± 31.64 g/m^2^, *p* = 0.0021) in the higher-UFR group. UFR was associated with LAD (OR: 3.6000, 95% CI: 1.3780–9.4053, *p* = 0.0089), LAVI (OR: 4.1067, 95% CI: 1.5478–10.8956, *p* = 0.0045), and LVMI (OR: 4.7619, 95% CI: 1.6825–13.4774, *p* = 0.0033). The frequency of ventricular and supraventricular arrhythmias was similar between the analyzed patient groups. **Conclusions:** Higher UFR was associated with structural changes in the left heart (LAVI, LAD, and LVMI), but not with other echocardiographic parameters or arrhythmias in hemodialysis patients.

## 1. Introduction

Nearly 4 million people worldwide receive renal replacement therapy (RRT), and approximately 69% of all RRT patients are treated via hemodialysis (HD) [1]. In patients treated with chronic hemodialysis, ultrafiltration is an effective method for removing excess extracellular fluid from the body. However, an inappropriate ultrafiltration rate may increase cardiovascular risk and mortality [2,3,4]. Recently, an observational study reported that patients with a higher proportion of HD sessions with ultrafiltration rate (UFR) > 13 mL/kg/h in their first 3 months of HD are at a higher risk of mortality, even when the average UFR over that period was <13 mL/kg/h [5].

Hemodialysis and different UFRs may induce structural and functional changes in the right and left heart [6,7]. Additionally, different UFRs may induce cardiac rhythm disturbances in hemodialysis patients [8].

The aim of this study was to determine the possible association of a higher UFR with morphological, functional, and electrophysiological changes in the left heart in hemodialysis patients treated via hemodiafiltration (HDF).

## 2. Materials and Methods

This work is a cross-sectional, observational, and clinical investigation conducted from 1 May 2024 until 31 December 2024. It included 75 hemodialysis patients, who were divided into two groups based on ultrafiltration rate: the higher-UFR group, ultrafiltration rate ≥ 10 mL/kg/h (*n* = 37); the lower-UFR group, ultrafiltration rate < 10 mL/kg/h (*n* = 38).

In this study, the inclusion criterion was being on hemodialysis for more than 5 years, and the exclusion criteria were as follows: (a) on hemodialysis for less than 5 years, (b) acute coronary syndrome, (c) severe systolic dysfunction, (e) cardiomyopathy, (d) severe valvular disease, (e) malignant tumors. This work was approved by the ethics committee of the institution where the study was realized. All patients were voluntarily included and provided written consent.

Data were collected through questionnaires and patient medical histories (age, sex, smoking, social status, comorbidities, etc.).

Patients underwent chronic conventional in-center hemodialysis 3 times a week at a clinic, with each session lasting 3 to 4 h, as well as hemodiafiltration (HDF). Participants were dialyzed on hemodialysis machines (Fresenius Medical Care 5008, Bad Homburg, Germany, and Baxter, Deerfield, IL, USA), with antithrombotic therapy (unfractionated or low-molecular-weight heparin). All machines had volumetric ultrafiltration control. The UFR was calculated according to the following formula:

$$\mathrm{UFR}\;(\mathrm{mL}/\mathrm{kg}/h)=\frac{\mathrm{Total}\;\mathrm{Fluid}\;\mathrm{to}\;\mathrm{Remove}\;(\mathrm{mL})}{\mathrm{Treatment}\;\mathrm{Time}\;(\mathrm{hours})\;\times \;\mathrm{Patient}\;\mathrm{Dry}\;\mathrm{Weight}\;(\mathrm{kg})\left[0.6.12\right]}$$

The central dialysis facility prepared the dialysis solution used during treatment, which contained the following: sodium, 140 mmol/L; potassium, 2.0 mmol/L; calcium, 1.25 mmol/L and 1.5 mmol/L; magnesium, 0.75 mmol/L; chlorides, 111.0 mmol/L; bicarbonates, 32.0 mmol/L; and glucose, 1.0 g/L.

### 2.1. Electrocardiographic and 48-Hour Holter Monitoring

Participants were fitted with a 12-lead BTL Holter unit. The recording covered one hemodialysis session and two non-dialysis days. Holter data were analyzed using the BTL software (2.41.474.0), with a subspecialist cardiologist reviewing the recordings.

The Holter recordings were used to analyze the duration and morphology of electrocardiographic changes, as well as the quantification and morphological characteristics of arrhythmic disorders and conduction abnormalities. Comparisons were made between electrocardiographic morphological changes and arrhythmic disorders during hemodialysis and the two days following treatment.

### 2.2. Echocardiographic Analysis

Cardiac morphological characteristics were analyzed using an ultrasound device (Vivid TB, serial number: 6008287WX0, GE HealthCare (formerly a division of General Electric, Beijing, China) before the start of hemodialysis treatment. A subspecialist cardiologist conducted echocardiographic analysis, assessing the dimensions of cardiac chambers, wall thickness, morphological and functional changes in wall motion and the valvular apparatus, systolic and diastolic function, and the cardiac mass index.

The echocardiographic measurements were taken according to “The 2025 American Society of Echocardiography (ASE) Guidelines for Left Ventricular (LV) Diastolic Function”.

The following indexing formulas were used:

(a) Body surface area (BSA) was calculated using the Mosteller Formula:

$$\mathrm{BSA}\left(m^{2}\right)=\sqrt{\frac{\mathrm{Height}\;(\mathrm{cm})\;\times \mathrm{Weight}\;(\mathrm{kg})}{3600}}$$

(b) Left ventricular mass index: (LVMI) (g/m^2^) = LVM/BSA.
(c) Left atrium volume index: (LAVI) (mL/m^2^) = LAV/BSA.

The left ventricular mass (LVM) was measured using a parasternal long-axis view at the level of the mitral valve leaflet tips during end-diastole (the onset of the QRS complex) and calculated using the Devereux formula:

LVM (g) = 0.8 × 1.04 × [(LVEDD + IVSd + PWd)^3^ − LVEDD^3^] + 0.6

LVEDD—left ventricular internal diameter at end-diastole; IVSd—interventricular septal thickness at end-diastole; PWd—posterior wall thickness at end-diastole.

E/e′ was calculated using the septal e′ measured via tissue Doppler imaging.

The left atrial volume (LAV) was measured at end-systole using the biplane method from the apical four- and two-chamber views and indexed to the body surface area as LVAI.

The left atrial anteroposterior diameter (LAD) was measured using a parasternal long-axis (PLAX) view at end-systole via 2D echocardiography or M-mode.

The echocardiographer was blinded to the UFR group and clinical data.

The primary outcomes in this study were the structural changes in the left heart, and the secondary outcomes were arrhythmias and functional parameters.

We used the deletion method to handle missing data; we removed the entire record of a patient if it was missing any value.

Continuous variables such as UFR, hs-cTnI, rest diuresis, weight loss on hemodialysis, and hypertension were dichotomized and transformed into categorical ones when needed for logical regression.

### 2.3. Statistical Analysis

All data collected during the study were analyzed using the JASP software package (version 0.18). Depending on the type and distribution of the data, the following statistical tests were used: Student’s *t*-test for independent samples to compare the means of continuous variables between two groups, provided the assumptions of normal distribution and homogeneity of variances were met; the Mann–Whitney U test as a nonparametric alternative when the distribution significantly deviated from normal or for ordinal data; Student’s *t*-test for dependent samples to compare values before and after dialysis within the same group; the chi-square test (χ^2^) to analyze associations between two categorical variables; and Fisher’s exact test when expected frequencies were less than 5 in more than 20% of cells. To assess normality and homogeneity of variance, the Shapiro–Wilk and Levene’s tests were used. Effect sizes were expressed using Cohen’s d (for *t*-tests) and the r coefficient (for Mann–Whitney tests) where applicable. Significance levels (*p* values) were interpreted with a conventional threshold of statistical significance at *p* < 0.05. Multivariable logistic regression analysis was performed to test the association of the UFR with the morphological and functional parameters of the left heart. All tests were two-tailed unless a one-tailed approach was clinically and methodologically justified.

## 3. Results

Statistically significant differences were not observed in baseline clinical characteristics of hemodialysis patients, except for the UFR expressed in mL/h (792.81 ± 75.86 vs. 601.35 ± 52.48 mL/h, *p* < 0.0001) and UFR per kg of body weight (12.06 ± 2.11 vs. 7.65 ± 1.07 mL/kg/h, *p* < 0.001), which were higher in patients with UFR ≥ 10 mL/kg/h (Table 1).

The morphological and functional parameters of the left heart (LH) of patients were assessed using echocardiographic measurements. Statistically significant higher values were observed for the left atrium diameter (LAD) (parasternal long axis—PLAX) (47.56 ± 4.82 vs. 44.75 ± 2.33 mm, *p* = 0.0019), the left atrial volume index (LAVI) (43.28 ± 7.13 vs. 36.43 ± 8.52 mL/m^2^, *p* = 0.0003), and the left ventricular mass index (LVMI) (138.47 ± 29.32 vs. 116.04 ± 31.64 g/m^2^, *p* = 0.0021) in patients with UFR ≥ 10 mL/kg/h compared to the lower-UFR group. Other morphological and functional parameters of the LH (end-diastolic diameter of the left ventricle (LV) (PLAX), thickness of the interventricular (IV) septum (parasternal short axis—PSAX), thickness of the back wall LV (PSAX), relative wall thickness of LV, the aortic annulus diameter (PLAX), the aortic bulb diameter (PLAX), and the ascending aorta diameter (PLAX)) were similar in analyzed patients (Table 2).

Multivariable logistic regression analysis showed that a higher ultrafiltration rate was associated with increased LAVI (OR: 4.1067, 95% CI: 1.5478–10.8956, *p* = 0.0045) (Table 3A), LAD (OR: 3.6000, 95% CI: 1.3780–9.4053, *p* = 0.0089) (Table 3B), and LVMI (OR: 4.7619, 95% CI: 1.6825–13.4774, *p* = 0.0033) (Table 4). The cut-off value for LAVI was 34 mL/m^2^: 48 (64%) patients had LAVI > 34 mL/m^2^ and 27 (36%) LAVI < 34 mL/m^2^. The cut-off value for LAD was 40 mm: 32 (42.7%) patients had LAD > 40 mm and 43 (57.3%) LAD < 40 mm. The cut-off value for LVMI was 115 g/m^2^: 55 (73.3%) patients had LVMI > 115 g/m^2^ and 20 (26.7%) LVMI < 115 g/m^2^. Other factors—hypertension, diabetes mellitus, duration of hemodialysis, hs-cTnI, rest diuresis, and weight loss on hemodialysis—were not associated with the parameters of cardiac structural remodeling of the left heart.

To evaluate heart rate indicators, temporal characteristics of the cardiac rhythm were analyzed via 48 h Holter monitoring. Based on the analysis, the values of any kind of ventricular or supraventricular arrhythmias, tachycardias, PQ interval, QTc interval, and absolute changes in the ST segment did not exhibit a statistically significant difference between the analyzed patient groups (Table 5).

## 4. Discussion

The results of this study show that a higher UFR is associated with parameters of cardiac structural remodeling, but not with parameters of systolic dysfunction of the left heart or the frequency of arrhythmias in hemodialysis patients.

Residual renal function (RRF) is the remaining ability of afflicted kidneys to remove water and metabolic waste in hemodialysis patients. The higher the RRF value, the lower the risk of mortality in hemodialysis patients [9]. Several studies have reported that higher residual diuresis indicates a higher residual glomerular filtration rate (GFR) in patients on hemodialysis [10]. In our study, residual diuresis did not statistically significantly differ in patients with UFR ≥ 10 mL/kg/h compared with those with UFR < 10 mL/kg/h.

Increased body weight is very important parameter for poor prognosis of clinical output in patients undergoing hemodialysis [11,12]. In our study, most patients in both groups had a normal BMI, with no significant difference between groups.

Several studies have reported that excessive interdialytic weight gain (IDWG) is associated with an increased cardiovascular risk and mortality [13,14,15]. In our study, interdialytic weight gain was in normal range and did not differ in analyzed patient’s group.

Standard hemodialysis (HD) and hemodiafiltration (HDF) are two methods for treating renal insufficiency, with HDF potentially offering better outcomes. Standard HD uses a low-flux membrane, while high-flux HD uses a membrane with larger pores. HDF combines diffusion (like HD) with convection (where fluid is infused) for improved solute removal, especially of larger molecules. High-volume HDF (HV-HDF) goes further, aiming for higher convection volumes; thus, it is increasingly considered the new standard of care in some regions [16]. In our study, patients were treated only with HDF, with all modalities of HDF, but no significant difference was observed in their frequency between the higher- and lower-UFR groups.

Based on earlier studies that have reported an association between weight-adjusted ultrafiltration rate (UFR) and cardiovascular risk [4,17,18], we divided patients into the higher-UFR group (≥10 mL/kg/h) and lower-UFR group (<10 mL/kg/h).

Echocardiographic parameters can be altered by renal failure itself and the hemodialysis process [19]. In our study, the left atrium diameter (LAD), the left atrial volume index (LAVI), and the left ventricular mass index (LVMI) were significantly larger in the higher-UFR group. UFR was associated with LAD, LAVI, and LVMI, while the other factors showed no statistical significance.

Kim et al. reported that a high UFR is a significant predictor and an increase of 1 mL/kg/h in the UFR is associated with a 22% higher risk of a worsening LAVI [20]. A high UFR (>10 mL/kg/h) might induce intradialytic hypotension (IDH), which is a common complication of hemodialysis, and IDH further increases LADI and LVMI [21]. In our study, IDH frequency did not differ in the higher- and lower-UFR patient groups, but LVMI was significantly higher in the former group, indicating a higher prevalence of hypertrophy in this group. Relative wall thickness was similar between groups, suggesting that hypertrophy is predominantly eccentric. A possible pathophysiological mechanism is cumulative hemodynamic stress.

Hickson et al. have reported that reduced left ventricular ejection fraction (LVEF) and left ventricular systolic dysfunction are independent risk factors for mortality in hemodialysis patients [22]. A higher UFR decreases LVEF [21]. In our study, no statistically significant difference in LVEF was observed between the higher- and lower-UFR groups.

The frequency of all types of arrhythmias was analyzed via 48 h Holter monitoring, and no statistically significant differences between the higher-UFR groups and lower-UFR groups. In our study, hemodialysis caused an increase in heart rate within groups, but the degree of this change (ΔHR) was not statistically different between patients with lower and higher UFRs. These findings suggest that heart rate change is a general response to treatment and not directly related to UFR.

In the USA, arrhythmias account for about 39% of total deaths in hemodialysis patients. Furthermore, ventricular tachycardia (VT) and ventricular fibrillation (VF) are the main causes of sudden cardiac death in patients with arrhythmia [1]. Holter studies covering 24 to 48 h have reported that a high UFR is associated with an increased frequency of arrhythmia and ECG changes during and after dialysis sessions [8,23].

Based on the analysis of ventricular and supraventricular arrhythmia and tachycardia parameters, no statistically significant differences were found between the higher- and lower-UFR groups in our study. These findings suggest that the occurrence of ventricular arrhythmias in this population is not significantly associated with differences in ultrafiltration burden or other analyzed characteristics of hemodialysis treatment.

Although certain forms of arrhythmias, such as trigeminy and quadrigeminy, were more frequent in the higher-UFR group, the differences were not statistically confirmed. The results indicate high inter-patient variability and the absence of a consistent pattern in the distribution of supraventricular arrhythmias in the context of the analyzed groups. None of the analyzed ECG parameters (PQ, QTc, ST) showed significant differences between groups. Specifically, the use of absolute values for ST deviations allowed for unbiased quantification of overall electrophysiological deviations, but there was no evidence of greater expression of these changes in either group. The results suggest similar electrophysiological stability of the myocardium in patients from both groups.

### Study Limitations

Despite a carefully designed methodology and comprehensive statistical analysis, this study has certain limitations that should be considered when interpreting the findings.

First, its cross-sectional study design is the primary methodological limitation. This design allows for the identification of associations but not causal relationships. Therefore, although statistical associations were found between ultrafiltration rate and certain cardiological parameters (e.g., LV mass, LA volume, dialysis duration), it cannot be definitively stated that UFR is a causal factor in their development.

Second, the sample and group sizes were limited. Although the number of participants met the minimum requirements for statistical analysis, the sample size may be insufficient to detect subtle but clinically significant differences in rare outcomes, such as the occurrence of ventricular tachycardias or more pronounced ST deviations.

Third is the variability in UFR and dialysis regimens. Ultrafiltration burden was operationalized as the average UFR value on the measurement day. However, in clinical practice, UFR values can vary significantly between treatments. The lack of longitudinal monitoring and average UFR values over a longer period limits our ability to assess the cumulative effect of ultrafiltration.

Fourth, confounders were under limited control. This study did not include additional potentially relevant variables such as IL-6. Due to the absence of these data, the effect of ultrafiltration alone is difficult to isolate. Electrolyte measurements were obtained but they were not included in the multivariable models. We acknowledge this as a potential source of residual confounding. The absence of statistically significant differences in bivariate comparisons alone is not sufficient justification for excluding potential confounders.

Fifth, there were some limitations in the technical interpretation of ECG parameters. Although 48 h Holter monitoring was used, analyses relied on the automatic detection of arrhythmias and intervals. This leaves room for technical errors in interpreting certain episodes, particularly supraventricular tachycardias and complex extrasystoles.

Sixth is potential survivor bias. Patients who have been on dialysis for many years and have been exposed to higher UFRs represent a group that has likely “survived” numerous complications. This bias may obscure the potentially harmful effect of high ultrafiltration, as the analysis included those who are most likely better adapted to dialysis stress.

Seventh, since no adjustment for multiple comparisons was performed, there is potential increased risk of type I error resulting from the number of comparisons conducted. Considering the number of predictors included in the analysis, there is potential risk for model instability/overfitting.

Despite these limitations, this study provides a significant contribution to our understanding of the relationship between ultrafiltration and cardiovascular stability in hemodialysis patients. Future studies with a longitudinal design, larger samples, and an expanded panel of biochemical and functional indicators are needed for more precise quantification of cardiovascular risks associated with ultrafiltration intensity.

## 5. Conclusions

A higher UFR is associated with structural changes in the left heart (LAVI, LAD, and LVMI), but no statistically significant association was observed between UFR and other echocardiographic parameters or arrhythmias in this sample of patients on hemodialysis.

## Figures and Tables

**Table 1:** Baseline clinical characteristics of hemodialysis patients and technical parameters of hemodialysis treatment.

Parameter	Higher-UFR Group (*n* = 37)	Lower-UFR Group (*n* = 38)	Cohen’s d	95% CI	*p*
Age (years)	57.23 ± 10.32	59.45 ± 13.28	0.186	−3.2634–7.7034	0.4224
BMI (kg/m^2^)	23.18 ± 1.63	24.39 ± 4.85	0.333	−0.4644–2.8844	0.1541
Male, *n* (%)	19 (51.4)	23 (60.5)			0.4304
Female, *n* (%)	18 (48.6)	15 (39.5)			
CRP (mg/L)	2.64 ± 0.62	2.38 ± 0.73	−0.383	−0.5721–0.0521	0.1011
Diabetes, *n* (%)	18 (48.6)	21 (55.3)		−15.2344–27.7644%	0.5641
Hypertension, *n* (%)	22 (59.5)	25 (65.8)		−15.0079–26.8938%	0.5753
Coronary artery disease, *n* (%)	19 (56.4)	16 (42.1)		−7.9916–34.7160%	0.2186
Congestive heart failure (CHF), *n* (%)	22 (59.5)	18 (47.4)		−10.0686–32.6693%	0.2969
Intradialytic hypotension (IDH), sessions with ≥1 IDH event, *n* (%)	7 (18.9)	4 (10.5)		−8.1495–24.9482%	0.3069
Antihypertensive medication, >1 type, *n* (%)	13 (35.1)	9 (23.7)		−9.0441% to 30.7334%	0.2815
Duration of HD treatment (month)	95.71 ± 98.24	84.37 ± 60.16	−0.140	−48.7178–26.0378	0.5473
Vascular access (AVF/Catheter)	34/3	30/8		−3.6300–29.0832%	0.1165
Rest diuresis/anuria	22/15	25/13		−15.0079–26.8938%	0.5753
Interdialytic weight gain (kg)	2.74 ± 0.55	2.46 ± 0.87	−0.384	−0.6160–0.0560	0.1010
Effective duration of the HD session (h)	3.85 ± 0.19	3.91 ± 0.22	0.292	−0.0347–0.1547	0.2107
Blood pump speed (mL/min)	252.15 ± 34.62	247.18 ± 29.63	−0.154	−19.7861–9.8461	0.5059
Dialysate flow rate (mL/h)	508.43 ± 58.25	503.96 ± 47.09	−0.085	−28.8149–19.8749	0.7155
Ultrafiltration rate (mL/h)	792.81 ± 75.86	601.35 ± 52.48	−2.942	−221.4112 to −161.5088	<0.0001
Ultrafiltration rate (mL/kg/h)	12.06 ± 2.11	7.65 ± 1.07	−2.647	−5.1769 to −3.6431	<0.0001

HD—hemodialysis; AVF—arteriovenous fistula; CRP—C reactive protein.

**Table 2:** Echocardiographic analysis of morphological and functional parameters of the left heart in hemodialysis patients.

Parameter	Higher- UFR Group (*n* = 37)	Lower- UFR Group (*n* = 38)	Cohen’s d	95% CI	*p* Value
Left atrium diameter (PLAX) (mm)	47.56 ± 4.82	44.75 ± 2.33	−0.745	−4.5451 to −1.0749	0.0019
Left atrial volume index (LAVI) (mL/m^2^)	43.28 ± 7.13	36.43 ± 8.52	−0.871	−10.4704 to −3.2296	0.0003
End-diastolic diameter of the LV (PLAX) (mm)	50.72 ± 6.02	49.69 ± 4.86	−0.189	−3.5446–1.4846	0.4170
Thickness of the IV septum (PSAX) (mm)	11.01 ± 1.93	10.59 ± 1.88	−0.220	−1.2968–0.4568	0.3429
Thickness of the back wall LV (PSAX) (mm)	10.37 ± 1.75	10.61 ± 2.02	0.127	−0.6307–1.1107	0.5845
Left ventricular mass index (g/m^2^)	138.47 ± 29.32	116.04 ± 31.64	−0.735	−36.4775 to −8.3825	0.0021
Relative wall thickness of LV	0.42 ± 0.14	0.41 ± 0.09	−0.085	−0.0640–0.0440	0.7132
E/e′	11.65 ± 7.30	10.83 ± 4.15	−0.139	−3.5436–1.9036	0.5503
Ejection fraction of the left ventricle	55.38 ± 9.13	57.26 ± 7.11	0.230	−1.8801–5.6401	0.3223
Diameter of the aortic annulus (PLAX) (mm)	26.25 ± 2.03	26.04 ± 2.73	−0.087	−1.3195–0.8995	0.7071
Diameter of the aortic bulb (PLAX) (mm)	33.17 ± 2.26	34.03 ± 1.57	0.443	−0.0335–1.7535	0.0590
Diameter of the ascending aorta (PLAX) (mm)	32.73 ± 4.03	33.12 ± 3.99	0.097	−1.4557–2.2357	0.6749

PLAX—parasternal long axis; PSAX—parasternal short axis; UFR—ultrafiltration rate.

**Table 3:** Multivariable logistic regression analysis of factors for (**A**) left atrial volume index (LAVI) and (**B**) left atrium diameter (LAD) in hemodialysis patients.

**(A) Left Atrial Volume Index (LAVI)**				
**Variable**	**Coding/** **Reference Category**	**Cut-Off Value/** **Threshold**	**OR (95% CI)**	***p*** **Value**
Age (years)	Continuous (per 5-year increase)	N/A	0.5859 (0.1724–1.9914)	0.3918
Diabetes	Categorical (no/yes)	N/A	1.7043 (0.6389–4.5467)	0.2869
Hypertension	Categorical (no/yes)	≤140 mm Hg >140 mm Hg	0.6487 (0.2486–1.6930)	0.3766
Duration of hemodialysis	Continuous (per 1-year increase)	N/A	0.5795 (0.2307–1.4561)	0.2458
Ultrafiltration rate	Categorical (low/high)	≤10 mL/kg/h >10 mL/kg/h	4.1067 (1.5478–10.8956)	0.0045
Rest diuresis	Categorical (low/normal)	≤250 mL/day >250 mL/day	1.4786 (0.5891–3.7112)	0.4049
Weight loss on hemodialysis	Categorical (normal/significant)	≤3.5 kg/session >3.5 kg/session	0.6964 (0.2522–1.9233)	0.4851
**(B) Left Atrium Diameter (LAD)**				
**Variable**	**Coding/** **Reference Category**	**Cut-Off Value/** **Threshold**	**OR (95% CI)**	***p* Value**
Age (years)	Continuous (per 5-year increase)	N/A	0.7258 (0.2249–2.3421)	0.5918
Diabetes	Categorical (No/yes)	N/A	0.8025 (0.2961–2.1752)	0.6653
Hypertension	Categorical (No/yes)	≤140 mm Hg >140 mm Hg	0.9565 (0.3797–2.4099)	0.9249
Duration of hemodialysis	Continuous (per 1-year increase)	N/A	2.2489 (0.8926–5.6660)	0.0856
Ultrafiltration rate	Categorical (low/high)	≤10 mL/kg/h >10 mL/kg/h	3.6000 (1.3780–9.4053)	0.0089
Rest diuresis	Categorical (low/normal)	≤250 mL/day >250 mL/day	0.5478 (0.2183–1.3750)	0.2000
Weight loss on hemodialysis	Categorical (normal/significant)	≤3.5 kg/session >3.5 kg/session	0.4926 (0.1944–1.2485)	0.1356

**Table 4:** Multivariable logistic regression analysis of factors for left ventricular mass index (LVMI) (g/m^2^) in hemodialysis patients.

Left Ventricular Mass Index (LVMI)				
Variable	Coding/ Reference Category	Cut-Off Value/ Threshold	OR (95% CI)	*p* Value
Age (years)	Continuous (per 5-year increase)	N/A	1.0579 (0.3884–2.8814)	0.9124
Diabetes	Categorical (no/yes)	N/A	2.3636 (0.9147–6.1080)	0.0758
Hypertension	Categorical (no/yes)	≤140 mm Hg >140 mm Hg	0.4701 (0.1818–1.2155)	0.1194
Duration of hemodialysis treatment	Continuous (per 1-year increase)	N/A	2.0513 (0.8110–5.1883)	0.1291
Ultrafiltration rate	Categorical (low/high)	≤10 mL/kg/h >10 mL/kg/h	4.7619 (1.6825–13.4774)	0.0033
Rest diuresis	Categorical (low/normal)	≤250 mL/day >250 mL/day	1.1060 (0.3566–3.4299)	0.8615
Weight loss on hemodialysis	Categorical (normal/significant)	≤3.5 kg/session >3.5 kg/session	1.7172 (0.3796–7.7684)	0.4826

**Table 5:** Arrhythmia in hemodialysis patients recorded over 48 h Holter monitoring according to ultrafiltration rate.

Parameter	Higher- UFR Group (*n* = 37)	Lower- UFR Group (*n* = 38)	Cohen’s d	95% CI	*p* Value
ΔHR (bpm)	75.21 ± 31.27	71.17 ± 23.67	−0.146	−16.7812 to 8.7012	0.5294
Monomorphic individual VES	668.32 ± 148.17	692.97 ± 194.36	0.142	−55.0417–104.3417	0.5395
Ventricular bigeminy	60.91 ± 71.05	62.23 ± 63.11	0.020	−29.5862–32.2262	0.9324
Ventricular trigeminy	221.48 ± 44.38	206.08 ± 57.74	−0.299	−39.1451–8.3451	0.2002
Ventricular quadrigeminy	67.37 ± 82.06	63.49 ± 31.23	−0.063	−32.3115–24.5515	0.7864
Ventricular cuplets	75.12 ± 25.32	70.09 ± 15.25	−0.241	−14.6197–4.5597	0.2993
Ventricular triplets	28.36 ± 17.24	24.37 ± 11.33	−0.274	−10.6864–2.7064	0.2389
Ventricular tachycardia	2.84 ± 1.83	2.27 ± 1.26	−0.364	−1.2914–0.1514	0.1196
Atrial extrasystoles (AES)	421.39 ± 130.62	432.18 ± 146.37	0.078	−53.1123–74.6923	0.7374
Supraventricular bigeminy	17.08 ± 6.36	20.15 ± 11.89	0.321	−1.3355–7.4755	0.1691
Supraventricular trigeminy	47.37 ± 13.56	49.34 ± 14.42	0.141	−4.4754–8.4154	0.5443
Supraventricular quadrigeminy	76.73 ± 16.76	78.92 ± 39.28	0.072	−11.7759–16.1559	0.7555
Supraventricular cuplets	11.38 ± 20.03	21.04 ± 25.66	0.419	−0.9528–20.2728	0.0738
Supraventricular triplets	3.32 ± 5.06	5.16 ± 9.72	0.237	−1.7407–5.4207	0.3092
Supraventricular tachycardia	12.80 ± 4.21	14.73 ± 5.80	0.380	−0.4076–4.2676	0.1042
PQ interval (msec)	169.23 ± 21.02	172.13 ± 24.31	0.127	−7.5706–13.3706	0.5826
QTc interval (msec)	457.49 ± 23.26	454.87 ± 26.77	−0.104	−14.1738–8.9338	0.6527
Absolute changes in the ST connector (expressed as average values in all drains over 48 h) (µV)(%)	28.23 ± 11.79	30.06 ± 10.22	0.166	−3.2436–6.9036	0.4745

UFR—ultrafiltration rate; VES—ventricular extrasystole; AES—atrial extrasystole; HR—heart rate.

## Data Availability

The original contributions presented in this study are included in the article. Further inquiries can be directed to the corresponding author.
